# The impact of surgeons’ visual angle misperception on acetabular cup positioning accuracy: a retrospective multicenter cohort study

**DOI:** 10.1097/JS9.0000000000003429

**Published:** 2025-09-10

**Authors:** Yuehao Hu, Minghao Jin, Yansong Qi, Yuanqing Mao, Zhenan Zhu, Xuqiang Liu, Jiying Chen, Zubing Mei, Huiwu Li, Zanjing Zhai

**Affiliations:** a Shanghai Key Laboratory of Orthopaedic Implants, Department of Orthopaedic Surgery, Shanghai Ninth People’s Hospital, Shanghai Jiao Tong University School of Medicine, Shanghai, People’s Republic of China; b Orthopedic Center (Sports Medicine Center), Inner Mongolia Autonomous Region People’s Hospital, Hohhot, People’s Republic of China; c Orthopedic Hospital, The First Affiliated Hospital, Jiangxi Medical College, Nanchang University, Jiangxi Province’s Artificial Joints Engineering and Technology Research Center, Nanchang, Jiangxi Province, People’s Republic of China; d Orthopaedic Department, Chinese PLA General Hospital, Beijing, People’s Republic of China; e Department of Anorectal Surgery, Shuguang Hospital Affiliated to Shanghai University of Traditional Chinese Medicine, Shanghai, China; f Anorectal Disease Institute of Shuguang Hospital, Shanghai, People’s Republic of China; g Department of Orthopedics, Shigatse People’s Hospital, Tibet, China

**Keywords:** abduction, anteversion, cup implantation, total hip arthroplasty, visual angle misperception

## Abstract

**Background:**

Precise acetabular cup placement in total hip arthroplasty (THA) heavily relies on surgeons’ visual judgment of angles. However, whether inherent visual angle misperception among surgeons affects surgical outcomes remains unclear. This study is the first to reveal that surgeons universally exhibit visual angle misperception, a key factor causing the cup implant positioning deviations in THA.

**Methods:**

We validated this phenomenon through three steps. Experimental validation: Five surgeons were asked completed 2D angle assessments to verify the visual angle misperception. Surgical simulation: A self-developed “Dynamic Angle-Measuring System (DAMS)” simulated cup implantation for assessing the impact of angle misperception on cup orientation. Clinical data: Retrospective analysis of 853 THA cases (683 patients) between 2015 and 2019 were collected for comparing mismatches between actual cup angles and surgeons’ target angles, and between experienced and junior surgeons.

**Results:**

All surgeons exhibited visual angle misperception in 2D and 3D angle assessments, with greater impact on anteversion than abduction angles. In in vitro simulations, discrepancies between actual and targeted anteversion angles were statistically significant (e.g., 16.8° vs. 20.0°, 22.6° vs. 25.0°; *P* < 0.05). These findings were corroborated in the in vivo data (e.g., 15.3° vs. 20.0°, 22.4° vs. 25.0°; *P* < 0.05), with an individualized fixed deviation among surgeons. Clinically, increased surgical experience only reduced the dispersion (SD value) without eliminating visual angle misperception bias.

**Conclusions:**

Our study is the first to provide that visual angle misperception is a hidden cause of cup positioning bias in THA, and experience alone cannot eliminate this bias causing by visual angle misperception, although experience reduced variability in placement. Recognizing and addressing this cognitive bias, and further targeted training to improve spatial perception, compensate inherent angle misperception bias, may lead to better patient outcomes.

## Introduction

Visual angle misperception – the human systematic tendency to overestimate or underestimate angular measurements – is a well-documented cognitive phenomenon in both two-dimensional (2D) and three-dimensional (3D) spaces^[1]^. Rooted in the brain’s reliance on empirical strategies to resolve ambiguous visual stimuli, this perceptual bias arises from the misperception of depth cues and spatial relationships, often shaped by prior experiences^[2-4]^. Studies across psychology and visual arts have demonstrated that observers consistently misjudge angles, with errors exacerbated in 3D contexts due to the added complexity of perspective and spatial disambiguation^[5-7]^. For instance, angular discrepancies in 3D assessments can exceed those in 2D tasks by up to 30%, underscoring the inherent limitations of human visual-spatial perception^[8]^. Despite its extensive characterization in non-clinical fields, the implications of visual angle misperception remain unexplored in surgical practice, especially orthopedic surgery, where precise spatial judgment is critical.

**HIGHLIGHTS**

- Visual angle misperception is a novel concept with significant implications in surgical procedures.
- Visual angle misperception leads to inaccurate cup implantation in THA, even among experienced surgeons.
- Anteversion angles are particularly affected by visual angle misperception, while abduction is less impacted.
- Increased surgical experience reduces variability but does not eliminate angle misperception bias.

Total hip arthroplasty (THA), a cornerstone procedure in orthopedic surgery, which exemplifies such precision-dependent challenge as precise placement of an acetabular cup within specific angular parameters^[9,10]^. With over 1.5 million annual procedures globally – a figure rising by 5% yearly. THA aims to restore function in patients with debilitating hip conditions such as osteoarthritis and femoral head necrosis^[11,12]^. Successful outcomes hinge on accurate acetabular cup positioning within the “safe zone”, yet even experienced surgeons frequently deviate from the safe zone^[13–16]^. Suboptimal cup placement correlates with severe complications, including dislocation, accelerated wear, and revision surgeries, which collectively burden healthcare systems and diminish patient quality of life^[17–19]^. While factors such as surgical position and approach, patient anatomy and BMI value, and pelvic tilt have been investigated as contributors to cause positioning inaccuracy^[20,21]^. A fundamental question still persists: why do surgeons, regardless of experience, struggle to achieve cup implantation within consistent angular accuracy?

**Figure 1.**
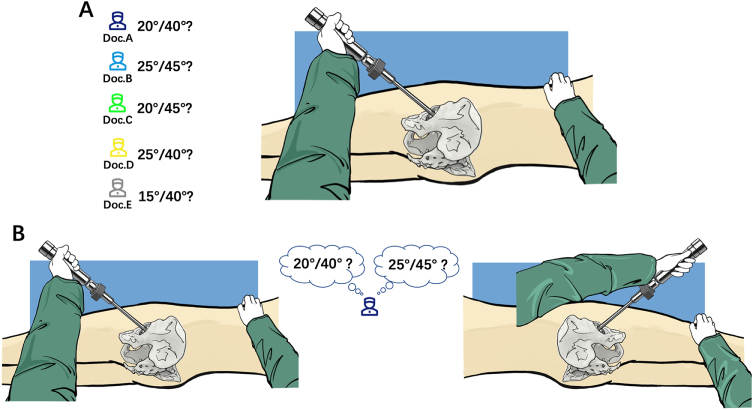
Diagram of angle misperception in THA. Surgeons’ visual-spatial perception could be different among different surgeons (A), as well as different positions (3D space) (B).

During THA procedure, the cup orientation involves surgeon’s complex visual-spatial awareness and angle judgment. The pelvis’s intricate 3D anatomy demands surgeons to interpret spatially dynamic relationships between anatomical landmarks while aligning the cup within narrow angular tolerances. This task is further complicated by perspective-dependent variations: identical cup angle may appear distinct depending on the surgeon’s vantage point, amplifying perceptual errors (Fig. 1). Thus, we hypothesize that visual angle misperception – a cognitive bias pervasive in non-surgical contexts – plays a pivotal role in THA cup implantation inaccuracies. We propose that surgeons’ inherent visual angle misperception, unaddressed by conventional training or experience, systematically biases cup orientation, leading to deviations between targeted and achieved positions.

Therefore, this study aims to bridge this knowledge gap by rigorously evaluating three hypotheses: (1) Whether visual angle misperception persists among surgeons across 2D and 3D assessments; (2) How does such misperception impacts acetabular cup positioning accuracy in THA surgeries; (3) Whether experience mitigates variability but fails to eliminate the underlying cognitive bias. By integrating experimental, simulated, and clinical data, we seek to establish visual angle misperception as a critical, previously unrecognized determinant of surgical precision, with implications for training and intraoperative guidance systems.

## Material and methods

### Study design and ethical approval

This multicenter retrospective cohort study was conducted across three medical centers and approved by the Ethics Committee of the institution. Informed consent was waived due to the anonymized nature of retrospective data. This cohort study has been reported in line with the STROCSS guidelines^[22]^.

### Surgeons and patients selection

**Figure 2.**
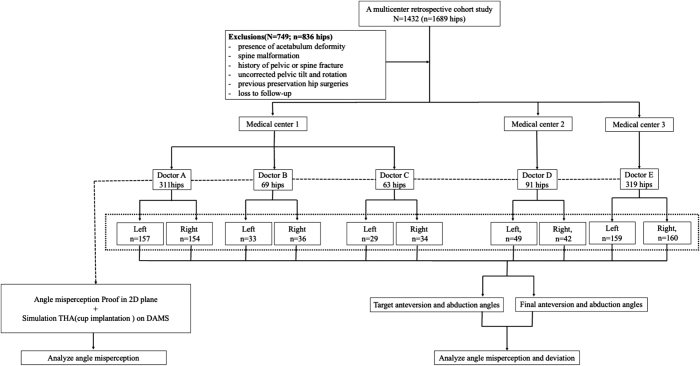
Flowchart depicting data selection and categorization of the study.

Five right-handed orthopedic surgeons (Doctors A–E) with varying experience levels were included. The surgeons aimed for their target cup orientation based on the “safe zone” theory, without considering combined anteversion. A total of 1432 consecutive patients (1689 hips) undergoing primary THA between January 2015 and July 2019 were retrospectively included. Inclusion criteria comprised: (1) aged 20–80 years; (2) underwent primary THA due to femoral neck fracture, femoral head necrosis, developmental hip dysplasia (Crowe I), or primary osteoarthritis; and (3) underwent surgery via the posterolateral approach with a cementless press-fit cup positioned at the surgeon’s target angle. Exclusion criteria included acetabular deformity, spinal malformation, history of pelvic or spinal fractures, uncorrected pelvic tilt or rotation, previous ipsilateral hip preservation surgeries (except arthroscopy), or loss to follow-up. Ultimately, 683 patients (853 hips) were retained for analysis (Fig. 2). Surgeries distribution per surgeon: Surgeon A: 311 THAs (232 patients, over 4 years); Surgeons B & C: 69 and 63 THAs (1 year); Surgeon D: 91 THAs (3 years); Surgeon E: 319 THAs (228 patients, over 4 years).

### Angle misperception assessment in 2D plane

To quantify inherent visual angle misperception, each surgeon performed standardized 2D angle-drawing tasks. Surgeons were instructed to draw 30° and 60° angles freehand on paper twice daily (8-hour intervals) over five consecutive days, yielding 20 angles per surgeon. Deviations from target angles were measured using digital protractor software, with temporal and spatial biases minimized through randomized task scheduling.

### In vitro simulation using dynamic angle-measuring system (DAMS)

**Figure 3.**
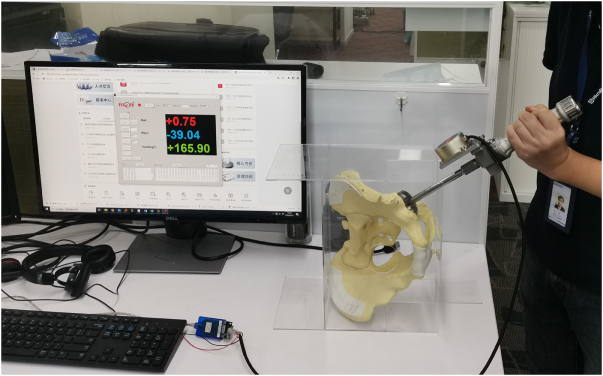
Dynamic angle-measuring system (DAMS) in vitro.

To isolate visual angle misperception’s impact on cup orientation, we developed a DAMS (Fig. 3) in collaboration with MicroPort (Suzhou, China). The system integrates: a synthetic pelvic model with adjustable 3D orientation; a cementless acetabular cup mounted on a jig with embedded gyroscope and accelerometer; real-time angle-tracking software calibrated to the anterior pelvic plane^[23,24]^. This system allowed for real-time monitoring of cup anteversion and abduction angles based on the anterior pelvic plane after calibration (the patent has been granted, Patent No. ZL202021966686.7). In the present study, prior to each use of the DAMS, its accuracy was verified with the “Skywalker” surgical robot system (model OSR-2000, MicroPort (Suzhou, China) OrthoBot Co. Ltd.) produced by the same company. Specifically, we first used the DAMS system to achieve the targeted prosthetic positioning (anteversion 20° and abduction 40°) on synthetic pelvic model. After that, we applied the Skywalker robot to detect the angles for each prosthetic positioning under identical conditions. The preliminary analyses of the mean absolute difference (MAD) and intraclass correlation coefficient (ICC) between the DAMS system and Skywalker robot showed a MAD of <1° for both anteversion and abduction and an ICC >0.95, indicating strong consistency.

To verify the influence of inherent visual angle misperception on cup orientation angles, we conducted in vitro surgical simulations of cup implantation, where cup orientation angles were recorded in real time. Five surgeons performed 20 simulated cup implantations per side (left/right) under standardized conditions. Confounding variables – including soft tissues, BMI, and pelvic tilt – were excluded to focus solely on perceptual errors.

### Surgical protocol

All THAs were performed via a posterolateral approach with patients in the lateral decubitus position under general anesthesia. The surgeons stood behind the patients during the procedure and cementless press-fit acetabular components and femoral stems were utilized. Surgeon-specific target angles are detailed in Table 1.

**Table 1:** General information of the five surgeons

	Medical center	THA experience (years)	Surgeons’ level	Period of data collection	Target cup orientation angles (anteversion/abduction)
Doctor A	I	>20	Senior	2015–2019	20°/40°
Doctor B	I	<20	Junior	2018–2019	25°/40°
Doctor C	I	<20	Junior	2018–2019	20°/40°
Doctor D	II	<20	Junior	2017–2019	15°/40°
Doctor E	III	>20	Senior	2016–2019	20°/40°

THA = total hip arthroplasty.

### Radiographic analysis

Postoperative cup orientation (anteversion and abduction angle) was measured using Einzel-Bild-Röntgen-Analysis (EBRA) software (University of Innsbruck, 2007), which has been validated for high accuracy and reliability^[25]^. Two blinded radiologists independently assessed all radiographs for angle measurement, with measurements repeated after 1 month to eliminate intraobserver and interobserver reliability.

### Outcome evaluation criteria and statistical analysis

Outcomes metrics included: deviation between targeted and achieved cup anteversion and abduction angles; percentage of outliers (as defined outside Lewinnek and Callanan safe zones); left-right asymmetry in angle distribution. All measurements were blinded to patient groups and study objectives to prevent bias.

Statistical analyses were conducted using SPSS (Version 24; SPSS Inc.) and Prism (Version 8; GraphPad Software Inc.). Results are presented as mean ± standard deviation. Wilcoxon and Mann–Whitney tests were employed to assess angle differences. Outlier percentages were calculated for each surgeon, and angle distribution was analyzed for standard deviation, skewness, and kurtosis using the student’s t-test. A two-tailed *P* <0.05 defined statistical significance. ICC values for reliability were categorized as follows: 0.81–1.00 (near-perfect reliability), 0.61–0.80 (strong reliability), 0.41–0.60 (moderate reliability), 0.21–0.40 (fair reliability), and 0–0.20 (poor reliability).

## Results

### Patient demographics

The multicenter retrospective study included 853 primary THAs (683 patients), with demographics and postoperative outcomes summarized in Table 2. For all radiographic measurements, intraobserver and interobserver reliability were high (ICC > 0.81). For statistical analyses, we used the mean values of the measured anteversion and abduction angles.

**Table 2:** Characteristics of the enrolled patients by five surgeons

	Doctor A	Doctor B	Doctor C	Doctor D	Doctor E
	(No. of patients = 232)	(No. of patients = 69)	(No. of patients = 63)	(No. of patients = 91)	(No. of patients = 228)
	(No. of hips = 311)	(No. of hips = 69)	(No. of hips = 63)	(No. of hips = 91)	(No. of hips = 319)
Sex					
Male	107	33	35	45	103
Female	125	36	28	46	115
Age* (yrs)	59.5 ± 13.6	61.5 ± 10.8	56.9 ± 13.5	62.7 ± 12.6	63.1 ± 10.2
BMI* (kg/m^2^)	24.1 ± 4.8	25.1 ± 4.9	25.9 ± 5.2	24.1 ± 4.5	25.2 ± 5.5
Hips					
Right	154	36	34	42	160
Left	157	33	29	49	159
Complications (dislocation, PJI, etc.)	0	0	0	0	0

* The values are given as the mean and standard deviation. BMI = body mass index. PJI = prosthetic joint infection.

### Validation of visual angle misperception among surgeons in vitro

**Figure 4.**
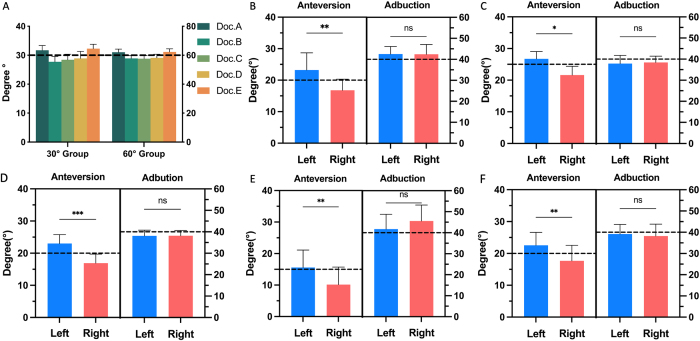
Difference between actual and targeted angles in 2D plane by Doctors A–E (A). Difference between targeted and measured angles in vitro by Doctors A–E (B–F). Black dotted line indicates the targeted angle. Besides, all surgeons achieved higher anteversion on the left side than right side (Doc. A: *P* = 0.002; Doc. B: *P* = 0.015; Doc. C: *P* < 0.001; Doc. D: *P* = 0.004; Doc. E: *P* = 0.002). The black dotted line indicates the targeted angles.

We compared the angles that surgeons drew on a 2D plane with the designated target angles (Fig. 4A) and confirmed that angle misperception exists and impacts surgeons’ inherent angle estimation, leading to biases that either exaggerate or underrate the intended angles.

Each surgeon (Doctors A–E) completed 20 cup implantation simulations on the DAMS for the in vitro verification of the influence of angle misperception on cup orientation. Significant discrepancies between targeted and achieved anteversion angles were observed in four surgeons: (Doc. A: *P* = 0.015, *P* < 0.001; Doc. B: *P* = 0.040, *P* = 0.004; Doc. C: *P* = 0.027, *P* = 0.014; Doc. E: *P* = 0.015, *P* = 0.008), while Doc. D exhibited a significant difference on one side only (*P* = 0.64, *P* = 0.001). No significant differences were observed between the targeted and achieved abduction angles in both sides (Fig. 4B-F). These in vitro results align closely with the performance of these surgeons in actual THA procedures, suggesting that angle misperception could consistently influence cup positioning.

### Clinical cup positioning accuracy in THA surgery

#### Targeted angles vs. actual angles

**Figure 5.**
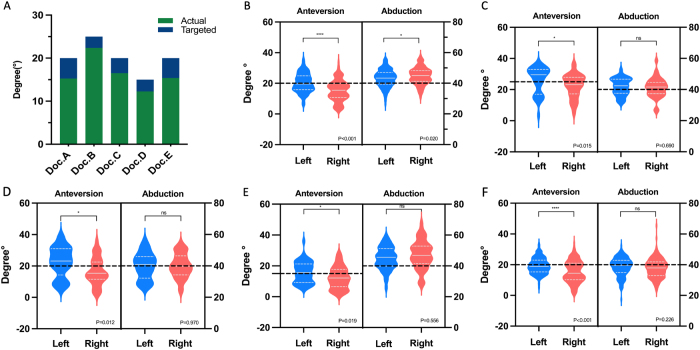
Difference between right actual and targeted cup anteversion (A). Final cup anteversion and abduction angles achieved by Doctors A–E (B–F); the black dotted line indicates the targeted angle; the white solid line indicates the mean actual angles; the white dotted line indicates the SD value of actual angles.

All surgeons set empirical target cup orientation angles without real-time adjustments during surgery. For all surgeons, achieved anteversion angles deviated significantly from the targeted angles, with trends that mirrored the in vitro results. In clinical cases, all five surgeons consistently undershot right-side anteversion targets (Doctors A–E: 15.3° ± 6.8 vs 20°, *P* < 0.001; 22.4° ± 8.1 vs 25°, *P* = 0.046; 16.5° ± 8.7 vs 20°, *P* = 0.024; 12.3° ± 7.1vs 15°, *P* = 0.016; 15.4° ± 7.0 vs 20°, *P* < 0.001), while there was no significant difference for left-side anteversion (Table 3 and Fig. 5A).

**Table 3:** Anteversion and abduction angles of the five surgeons

	N (R/L)	Anteversion (°)				Abduction (°)			
		Right	P*	Left	P*	Right	P*	Left	P*
**Doctor A** (20°/40°)	154/157	15.27 ± 6.81	<0.001	20.27 ± 6.19	0.588	44.43 ± 5.50	<0.001	43.25 ± 5.73	<0.001
**Doctor B** (25°/40°)	36/33	22.38 ± 8.10	0.046	26.02 ± 8.55	0.498	41.20 ± 6.28	0.257	41.77 ± 5.46	0.071
**Doctor C** (20°/40°)	34/29	16.50 ± 8.65	0.024	22.46 ± 9.62	0.180	40.10 ± 7.66	0.938	39.98 ± 8.43	0.991
**Doctor D** (15°/40°)	42/49	12.26 ± 7.08	0.016	15.76 ± 6.86	0.440	47.23 ± 9.07	<0.001	44.92 ± 7.31	<0.001
**Doctor E** (20°/40°)	160/159	15.38 ± 7.02	<0.001	19.25 ± 5.18	0.071	37.80 ± 6.31	<0.001	38.58 ± 5.99	0.003

* Comparison between the targeted and actual angles. R/L = right/left.

#### Left-right asymmetry of cup orientation angles

Left-side anteversion was systematically higher than right-side values across all surgeons (Δ_Doc.A_ = 5.0°, *P* < 0.01, Δ_Doc.B_ = 6.4°, *P* = 0.015, Δ_Doc.C_ = 7.8°, *P* = 0.012, Δ_Doc.D_ = 3.5°, *P* = 0.019, Δ_Doc.E_ = 4.4°, *P* < 0.01, Δ = Left Anteversion-Right Anteversion; Fig. 5B-F), while abduction angles remained symmetric in most surgeons.

#### Safe zones and distribution of cup orientation angles

**Figure 6.**
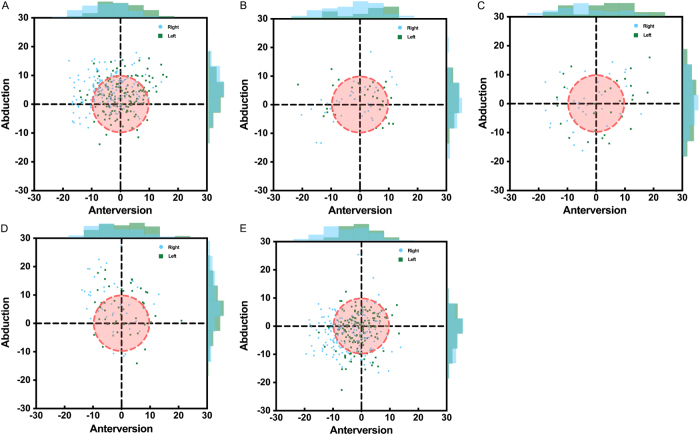
Distribution of cup anteversion and abduction (A–E). The red dotted line circle indicates the 10° boundary around the target angles. The bar charts surrounded showed the distribution of angular data.

The distribution of cup anteversion and abduction angles for all five surgeons, across both sides, is shown in Fig. 6 and Table 4. Doctors A–E placed 61.4%, 60.9%, 31.5%, 45.1%, and 67.4% of cups within a 10° boundary around the target angles, respectively (Table 4). Even experienced surgeons had cup placement accuracies within the safe zone less than 70% of cases. Based on experience (Table 1), we divided surgeons into Senior (Doctors A and E) and Junior (Doctors B, C, and D) groups. Comparing the dispersion of anteversion and abduction angles, a significant difference in anteversion deviation was found between these groups (SD: 6.50 vs 8.32, *P* = 0.019), indicating that senior surgeons had less angle deviation and better accuracy. However, neither group achieved consistently high accuracy, suggesting that although surgical experience reduces variation, it does not counteract angle misperception.

**Table 4:** The concentrate distribution (10° as the boundary) and the discrete distribution of anteversion and abduction (Doctors A–E)

	Doctor A		Doctor B		Doctor C		Doctor D		Doctor E	
	Inner	Outlier	Inner	Outlier	Inner	Outlier	Inner	Outlier	Inner	Outlier
**Left (N/%)**	110 (70.1%)	47 (29.9%)	19 (57.6%)	14 (42.4%)	8 (27.6%)	21 (72.4%)	24 (49.0%)	25 (51.0%)	128 (80.5%)	31 (19.5%)
**Right (N/%)**	81 (52.6%)	73 (47.4%)	23 (63.9%)	13 (26.1%)	15 (44.1%)	19 (55.9%)	17 (40.1%)	25 (59.9%)	87 (54.4%)	73 (45.6%)
**Total (N/%)**	191 (61.4%)	120 (38.6%)	42 (60.9%)	27 (39.1%)	23 (31.5%)	40 (68.5%)	41 (45.1%)	50 (54.9%)	215 (67.4%)	104 (32.6%)
	**An (L/R)**	**Ab (L/R)**	**An (L/R)**	**Ab (L/R)**	**An (L/R)**	**Ab (L/R)**	**An (L/R)**	**Ab (L/R)**	**An (L/R)**	**Ab (L/R)**
**SD**	6.19/6.81	5.73/5.50	8.55/8.10	5.46/6.28	9.62/8.65	8.43/7.77	6.87/7.08	7.32/9.07	5.18/7.02	5.99/6.31
**Skewness**	0.155/0.132	−0.221/-0.033	−0.870/-0.235	−0.198/-0.062	−0.257/0.330	−0.030/-0.041	0.443/0.363	−0.233/-0.069	0.202/0.100	−0.595/0.517
**Kurtosis**	−0.417/-0.416	<-0.001/-0.206	−0.200/-0.498	−0.879/1.338	−0.954/-0.503	−0.920/-0.579	−0.118/-0.648	−0.191/-0.171	−0.156/-0.679	0.298/1.636

An = anteversion; Ab = abduction; SD = standard deviation; L/R = left/right.

## Discussion

### Key findings

This study is the first to identify inherent visual angle misperception – a systematic cognitive bias in spatial angle judgment – as a critical determinant of acetabular cup positioning errors in THA surgery, particularly in anteversion angles. All surgeons exhibited visual angle misperception in both 2D assessments and 3D simulations, leading to significant deviations between targeted and actual cup anteversion angles. Notably, senior surgeons demonstrated comparable absolute deviations and less variation in cup positioning than their junior counterparts, suggesting that experience may fails to eliminate this perceptual bias causing by visual angle misperception, and underscoring that experience mitigates random error but not systematic bias.

## Mechanisms of visual angle misperception

Visual angle misperception arises from the brain’s reliance on heuristic strategies to resolve ambiguous spatial information, a phenomenon well-documented in psychology and visual arts^[26–28]^. In surgical contexts, this bias is exacerbated by the pelvis’s intricate 3D anatomy, where identical angles appear distinct depending on the surgeon’s vantage point (Fig. 1). Our findings align with Harris *et al*^[29]^, who attributed orientation biases to proprioceptive limitations that persist across perceptual settings. The persistence of misperception among experienced surgeons suggests that visual-spatial judgment in THA is constrained by intrinsic cognitive limitations rather than technical skill deficits.

### Comparison with prior research

While previous studies attributed cup positioning errors to external factors like bilateral position or pelvic tilt^[30,31]^. However, these studies did not fully account for the intrinsic perceptual biases that we propose. Instead, they attributed discrepancies to external factors like maneuverability, which we found unconvincing based on our data. Our study provides a cognitive explanation that bridges this gap, showing that inherent visual angle misperception could play a central role in these previously reported orientation discrepancies^[32,33]^. Similarly, while earlier work didn’t link the surgical experience to reduced variability^[34]^, our study uniquely demonstrates that experience does not address the underlying cognitive bias. This paradigm shift – from external to intrinsic factors – provides a unifying explanation for longstanding inconsistencies in THA outcomes.

## Clinical implications

Recognizing the impact of inherent visual angle misperception on cup positioning in THA has immediate implications for surgical practice and training. Given that accurate cup placement is critical to reducing complications like dislocation, wear, and revision surgeries, addressing this cognitive bias could lead to improved surgical outcomes. Reliance solely on surgical experience may be insufficient to achieve consistent cup positioning within the safe zone, instead incorporating simulation-based training that targets visual angle misperception could achieve more accurate visual-spatial judgment. For junior surgeons, targeted training could reduce variability, while seniors may benefit from tools that address ingrained biases. Besides, specific intraoperative technologies (e.g., navigation systems, surgical robot) that may help mitigate misperception during surgery. The high costs associated with robotic-assisted technologies and navigation systems may limit their clinical dissemination. However, in this study, training programs for pre-operative simulation correction based on visual angle misperception are more likely to become a cost-effective clinical paradigm for achieving precise intraoperative cup placement in THAs with great potential of transformation chain from basic cognitive mechanisms to clinical development in the field of surgical precision.

Meanwhile, AR-integrated angle calibration training system could be developed, in which the DAMS platform will be augmented with an AR module that projects real-time angular reference lines onto the simulated acetabular component. Surgeons will receive immediate AR-guided cues during repetitive practice sessions, allowing for iterative adjustment of their visual angle perception until target angles are consistently achieved. The intraoperative feedback tool linkage could also be developed to outline a planned integration pathway between preoperative DAMS training data and an intraoperative visual feedback system. This system will display real-time angular parameters (compared against surgeon-specific target ranges established during DAMS training) via a heads-up display, providing corrective prompts when deviations exceed predefined thresholds.

### Strengths and limitations

Our study possesses several key strengths. First, we offer a novel cognitive perspective, which firstly links visual angle misperception, a well-established psychological phenomenon, to THA surgical precision. This perspective is groundbreaking, as prior researches largely focused on external factors, while we highlight an intrinsic perceptual bias within surgeons, potentially applicable across other precision-dependent surgeries. Second, our comprehensive methodology with combined 2D experimental validation, in vitro simulations (DAMS), and multicenter clinical data to isolate and quantify perceptual bias causing by visual angle misperception. Third, our multicenter design, involving surgeons across experience levels and institutions enhances translational relevance. To further enhance understanding, we included an animated Supplemental Digital Content Video 1 (available at: http://links.lww.com/JS9/F104), which visually clarifies visual angle misperception, making our findings accessible to a broader audience, including readers and reviewers.

Despite these strengths, the study has several limitations. First, all THA procedures were restricted to the posterolateral approach by right-handed surgeons, which may limit generalizability to other surgical techniques and patient positions, as well as the handedness of the surgeons. The findings of this study might differ with the use of alternative surgical approaches, as well as left-handed surgeons. Additionally, the retrospective design introduces potential selection bias, while prospective trials are needed to validate causality. Furthermore, the data were collected within a single country, and cross-cultural validation is warranted. Hence, we intend to expand multidimensional generalizability validation studies, with multi-approach comparison, left-handed subgroup and cross-ethnic cohort expansion for theoretical generalizability, as well incorporating clinical outcome measures (e.g., Harris Hip Score, HHS) and patient-reported outcomes to systematically evaluate the clinical impact of angular deviations resulting by misperception. Besides, intervention strategies of THA surgery followed by the DAMS-system based simulation correction will be carried out for the translation of research findings into clinical practice.

## Conclusion

This study establishes inherent visual angle misperception as a pivotal, previously unrecognized factor for cup positioning accuracy in THAs. We demonstrated that this perceptual bias leads to deviations in cup anteversion angles, with senior surgeons showing less dispersion but still unable to mitigate the misperception effect. By bridging cognitive psychology and surgical science, we provide a framework for rethinking training paradigms and technological interventions. Future studies should focus on debiasing strategies – such as adaptive simulations and augmented reality – to transcend the limitations of human visual angle misperception, ultimately improving the precision and reliability of cup placement in THA.

## Data Availability

All data included in this study are available upon reasonable request by contacting the corresponding author.

## Research registration unique identifying number (UIN)

Research Resitry (unique identifying number or registration ID: researchregistry7972).

## References

[1] Hammad S Juricevic I Rajani S. Angle illusion on a picture’s surface. Spat Vis 2008;21:451–62. 18534114 10.1163/156856808784532554

[2] Gregory RL. Distortion of visual space as inappropriate constancy scaling. Nature 1963;199:678–80. 14074555 10.1038/199678a0

[3] Nundy S Lotto B Coppola D. Why are angles misperceived? Proc Natl Acad Sci U S A 2000;97:5592–97. 10805814 10.1073/pnas.97.10.5592 PMC25873

[4] Williams SM McCoy AN Purves D. The influence of depicted illumination on brightness. Proc Natl Acad Sci U S A 1998;95:13296–300. 9789082 10.1073/pnas.95.22.13296 PMC23788

[5] Ostrofsky J Kozbelt A Cohen DJ. Observational drawing biases are predicted by biases in perception: empirical support of the misperception hypothesis of drawing accuracy with respect to two angle illusions. Q J Exp Psychol (Hove) 2015;68:1007–25. 25405522 10.1080/17470218.2014.973889

[6] Blakemore C Carpenter RH Georgeson MA. Lateral inhibition between orientation detectors in the human visual system. Nature 1970;228:37–39. 5456209 10.1038/228037a0

[7] Gonzales RA Ferns G Vorstenbosch MATM. Does spatial awareness training affect anatomy learning in medical stu dents? Anat Sci Educ 2020;13:707–20. 32048478 10.1002/ase.1949

[8] Coppola DM Purves HR McCoy AN. The distribution of oriented contours in the real world. Proc Natl Acad Sci U S A 1998;95:4002–06. 9520482 10.1073/pnas.95.7.4002 PMC19952

[9] Pivec R Johnson AJ Mears SC. Hip arthroplasty. Lancet 2012;380:1768–77. 23021846 10.1016/S0140-6736(12)60607-2

[10] Sloan M Premkumar A Sheth NP. Projected volume of primary total joint arthroplasty in the U.S., 2014 to 2030. J Bone Joint Surg Am 2018;100:1455–60. 30180053 10.2106/JBJS.17.01617

[11] Lewinnek GE Lewis JL Tarr R. Dislocations after total hip-replacement arthroplasties. J Bone Joint Surg Am 1978;60:217–20. 641088

[12] Callanan MC Jarrett B Bragdon CR. The John Charnley award: risk factors for cup malpositioning: quality improvement through a joint registry at a tertiary hospital. Clin Orthop Relat Res 2011;469:319–29. 20717858 10.1007/s11999-010-1487-1 PMC3018230

[13] Beamer BS Morgan JH Barr C. Does fluoroscopy improve acetabular component placement in total hip arthroplasty? Clin Orthop Relat Res 2014;472:3953–62. 25238804 10.1007/s11999-014-3944-8 PMC4397754

[14] Barrack RL Krempec JA Clohisy JC. Accuracy of acetabular component position in hip arthroplasty. J Bone Joint Surg Am 2013;95:1760–68. 24088968 10.2106/JBJS.L.01704

[15] Domb BG El Bitar YF Sadik AY. Comparison of robotic-assisted and conventional acetabular cup placement in THA: a matched-pair controlled study. Clin Orthop Relat Res 2014;472:329–36. 23990446 10.1007/s11999-013-3253-7 PMC3889439

[16] Sariali E Boukhelifa N Catonne Y. Comparison of three-dimensional planning-assisted and conventional acetabular cup positioning in total hip arthroplasty: a randomized controlled trial. J Bone Joint Surg Am 2016;98:108–16. 26791031 10.2106/JBJS.N.00753

[17] Biedermann R Tonin A Krismer M. Reducing the risk of dislocation after total hip arthroplasty: the effect of orientation of the acetabular component. J Bone Joint Surg Br 2005;87-B:762–69. 10.1302/0301-620X.87B6.14745 15911655

[18] Harrison CL Thomson AI Cutts S. Research synthesis of recommended acetabular cup orientations for total hip arthroplasty. J Arthroplasty 2014;29:377–82. 23958234 10.1016/j.arth.2013.06.026

[19] Leslie IJ Williams S Isaac G. High cup angle and microseparation increase the wear of hip surface replacements. Clin Orthop Relat Res 2009;467:2259–65. 19363640 10.1007/s11999-009-0830-x PMC2866926

[20] Buckland AJ Vigdorchik J Schwab FJ. Acetabular anteversion changes due to spinal deformity correction: bridging the gap between hip and spine surgeons. J Bone Joint Surg Am 2015;97:1913–20. 26631991 10.2106/JBJS.O.00276

[21] Grammatopoulos G Pandit HG da Assunção R. The relationship between operative and radiographic acetabular component orientation: which factors influence resultant cup orientation? Bone Joint J 2014;96-b:1290–97. 25274911 10.1302/0301-620X.96B10.34100

[22] Agha RA Mathew G Rashid R. Revised strengthening the reporting of cohort, cross-sectional and case-control studies in surgery (STROCSS) guideline: an update for the age of artificial intelligence. Prem J Sci 2025;10:100081.

[23] Allseits E Agrawal V Lučarević J. A practical step length algorithm using lower limb angular velocities. J Biomech 2018;66:137–44. 29198369 10.1016/j.jbiomech.2017.11.010

[24] Staab W Hottowitz R Sohns C. Accelerometer and gyroscope based gait analysis using spectral analysis of patients with osteoarthritis of the knee. J Phys Ther Sci 2014;26:997–1002. 25140082 10.1589/jpts.26.997 PMC4135223

[25] Krismer M Bauer R Tschupik J. EBRA: a method to measure migration of acetabular components. J Biomech 1995;28:1225–36. 8550641 10.1016/0021-9290(94)00177-6

[26] Kincade S Wilson AE. Effects of method, orientation, and size of angle on the Ponzo illusion. Percept Mot Skills 2000;91:837–47. 11153859 10.2466/pms.2000.91.3.837

[27] Lotto RB Purves D. The effects of color on brightness. Nat Neurosci 1999;2:1010–14. 10526341 10.1038/14808

[28] Purves D Shimpi A Lotto RB. An empirical explanation of the cornsweet effect. J Neurosci 1999;19:8542–51. 10493754 10.1523/JNEUROSCI.19-19-08542.1999 PMC6783017

[29] Fraser LE Harris LR. Perceived finger orientation is biased towards functional task spaces. Exp Brain Res 2016;234:3565–74. 27534861 10.1007/s00221-016-4752-z

[30] Pennington N Redmond A Stewart T. The impact of surgeon handedness in total hip replacement. Ann R Coll Surg Engl 2014;96:437–41. 25198975 10.1308/003588414X13946184902488 PMC4474195

[31] Song X Ni M Li H. Is the cup orientation different in bilateral total hip arthroplasty with right-handed surgeons using posterolateral approach? J Orthop Surg Res 2018;13:123. 29792206 10.1186/s13018-018-0789-y PMC5967059

[32] Bosker BH Verheyen CCPM Horstmann WG. Poor accuracy of freehand cup positioning during total hip arthroplasty. Arch Orthop Trauma Surg 2007;127:375–79. 17297597 10.1007/s00402-007-0294-y PMC1914284

[33] Minoda Y Kadowaki T Kim M. Acetabular component orientation in 834 total hip arthroplasties using a manual technique. Clin Orthop Relat Res 2006;445:186–91. 16467620 10.1097/01.blo.0000201165.82690.f8

[34] Leichtle U Gosselke N Wirth C. Radiologic evaluation of cup placement variation in conventional total hip arthroplasty. Rofo 2007;179:46–52. 17203443 10.1055/s-2006-927085

